# Comparative Analysis of Gel Properties of Sodium Citrate-Treated Giant Squid and Pork for Surimi Production

**DOI:** 10.3390/gels11110893

**Published:** 2025-11-06

**Authors:** Hongliang Mu, Zufang Wu

**Affiliations:** College of Food Science and Engineering, Ningbo University, Ningbo 315832, China

**Keywords:** *Dosidicus gigas*, rheological properties, gel properties, microstructure, WHC, LF-NMR

## Abstract

The giant squid, despite its abundance as a resource, is underutilized for surimi production due to its distinctive odor and poor gel-forming ability. While soaking (e.g., in sodium citrate) can alleviate the odor, its impact on the gel properties remains unclear. This study employed a comparative approach using pork, a benchmark for high-quality gels, to critically evaluate the gel properties of deodorized giant squid. The rheological, textural, and microstructural properties, as well as the water-holding capacity and water distribution, of squid (after sodium citrate soaking) and pork gels were compared. The results demonstrated that the squid gels exhibited a significantly lower storage modulus and higher tan δ value than pork gels, indicating inferior rheological properties. After cooking, the squid gel exhibited a bent shape and markedly lower hardness (approximately 259.78 g) and chewiness (approximately 226.09 g) compared to the pork gels (approximately 3305.92 g and 2781.27 g, respectively). Microstructurally, the squid gels presented a coarse, porous, and discontinuous network with larger pores, contrasting sharply with the fine, dense, and uniform matrix of the pork gels. Correspondingly, the squid gels had inferior water-holding capacity and a higher proportion of free water. This comparison demonstrates that the gel from sodium citrate-soaked giant squid is weak. More importantly, it provides mechanistic insights by highlighting the specific structural and hydration deficiencies responsible for its poor performance. The findings underscore that targeted strategies to modify the protein network are necessary for the effective utilization of giant squid in surimi production.

## 1. Introduction

Surimi can be produced from a variety of species, such as hairtail (*Trichiurus lepturus*) [1], silver carp (*Hypophthalmichthys molitrix*) [2], tilapia (*Oreochromis niloticus*) [3], and shrimp (*Litopenaeus vannamei*) [4]. The giant squid (*Dosidicus gigas*) represents a potential alternative source for surimi production [5]. However, its utilization faces two primary challenges: firstly, protein degradation during post-capture storage can adversely impact gel performance [6]; secondly, its unique odor may limit consumer acceptance [7].

To address the issue of gelation, various strategies have been explored. The addition of substances such as laver powder [8], organic salts [9], egg white protein [10], and transglutaminase [11] has proven effective. Processing conditions also play a critical role; for instance, high-pressure treatment (300 MPa/30 min/15 °C) was shown to enhance both the “suwari” and heated gels’ mechanical and water-binding properties [11]. Furthermore, the acid washing method was found to preserve myofibrillar protein functionality better than isoelectric precipitation, resulting in a superior gel structure [12].

Concurrently, studies on odor removal have identified soaking as a common deodorization technique. Soaking in solutions like sodium citrate, sodium tripolyphosphate, glucose, sodium carbonate, and sodium bicarbonate can improve sensory qualities, with 2% sodium citrate for 15 h reported as optimal [13]. A two-stage soaking process (first in 1% sodium carbonate, then in composite phosphate) has been developed to enhance quality [14]. Separately, ultrasonic treatment has been shown to be an effective method for accelerating the deodorization process [15].

Critically, mitigating undesirable flavors is a prerequisite for improving gel properties. However, it remains unclear whether the giant squid used in the aforementioned gel enhancement studies underwent any pre-treatment for deodorization. This omission could confound the interpretation of results related solely to gel improvement.

In contrast, pork is renowned for its favorable gelation properties [16] and is widely consumed, often processed into various fermented products like sausages [17,18,19]. While methods to enhance pork gel properties (e.g., adding κ-carrageenan/non-meat proteins [20] or ultrasonic treatment [21]) have been studied, its inherent gelation ability is well established [16].

Therefore, using pork as a reference material provides a clear target for what constitutes a high-quality gel. By systematically contrasting the gelation behavior and resulting structure of giant squid against this high-standard benchmark, we can gain unique insights into the specific functional deficiencies of giant squid. This approach moves beyond merely improving squid gel strength and aims to elucidate the underlying reasons for its poor performance by highlighting the contrasts with a proven gel-forming system.

## 2. Results and Discussion

### 2.1. Comparison of Rheological Properties of Giant Squid and Pork

**Figure 1:**
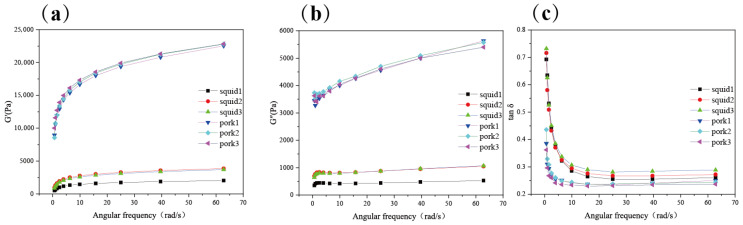
The storage modulus (**a**), loss modulus (**b**), and tan δ (**c**) of giant squid and pork (*n* = 3).

Firstly, the rheological properties of pork and giant squid were compared. Storage modulus (G′) and loss modulus (G″) describe the elastic and viscous properties of samples, respectively [22]. The rheology results indicated that during the frequency test, the storage modulus of the giant squid and pork was always higher than the loss modulus (Figure 1a,b,c), suggesting that in both pork and squid, elasticity is the main property [23]. In food, for example, in the gluten network, a higher G′ is related to an enhancement of cross-linking, while a lower G′ is related to less cross-linking and a weaker structure [22]. In the present study, the G′ of squid was lower than that of pork (Figure 1a), indicating that the network of squid was poorer than pork. Tan δ, the ratio of G″ and G′, could reflect the firmness of samples [22]. For dough, a lower tan δ value indicates a firm and rigid sample, while a higher value indicates a soft and elastic one [22]. From Figure 1c, it is evident that the tan δ of squid is higher than that of pork, implying that squid is less solid-like. The rheological results showed that the gel ability of the soaked squid samples was lower than that of pork.

### 2.2. Comparison of Appearance, Texture, and Microstructure of Giant Squid and Pork

**Figure 2:**
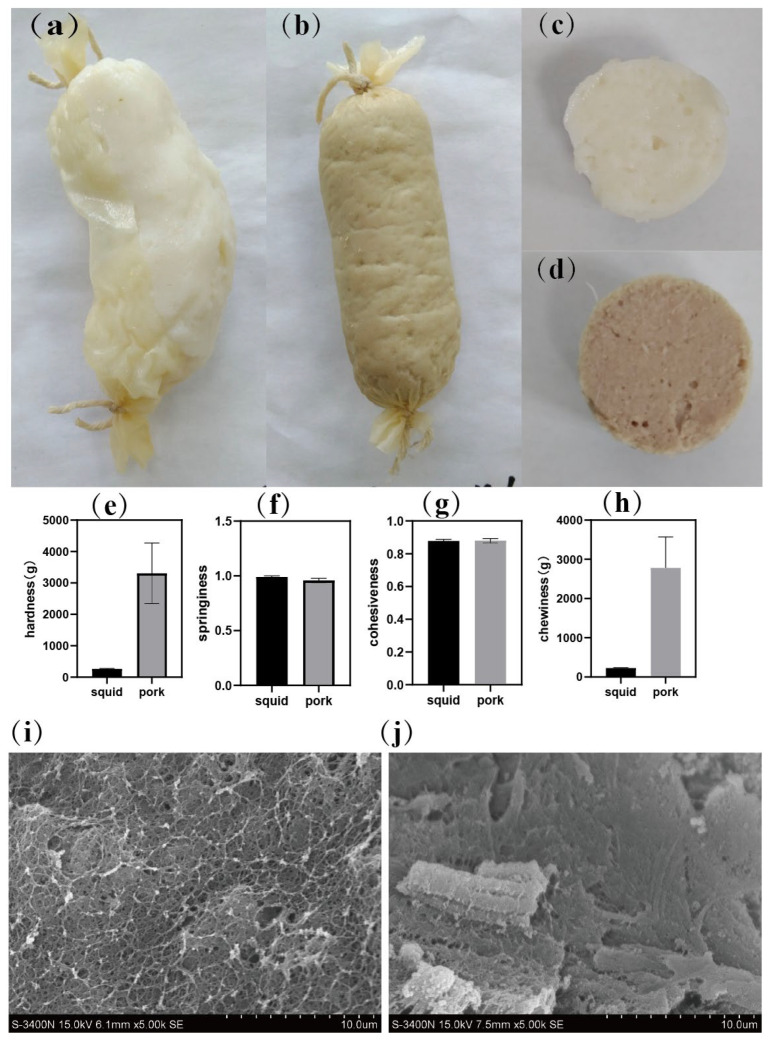
The appearance (**a**–**d**), texture (**e**–**h**), and microstructure (**i**,**j**) of giant squid and pork. (**a**,**c**,**i**) squid sample; (**b**,**d**,**j**) pork sample.

The appearance and texture of cooked samples of giant squid and pork were compared. Figure 2a–d show the appearance of pork and squid after cooking. It was easy to see that after cooking, the squid sample’s casing was prone to damage and the shape was also prone to bending. Correspondingly, the morphology of the pork sample was intact. In addition, the squid sample had larger holes than the pork. The appearance of the giant squid might affect its acceptance by consumers.

The texture of squid and pork was also compared. The results (Figure 2e–h) show that the hardness and chewiness of pork (approximately 3305.92 g and 2781.27 g, respectively) were significantly higher than those of squid (approximately 259.78 g and 226.09 g, respectively) (*p* < 0.05), while the springiness was significantly lower than the squid samples (*p* < 0.05), and no significant difference was found in terms of cohesiveness between the pork and squid. The increase in hardness seen in noodles supplemented with soy protein is believed to be related to the network between starch and protein [24]. The present results suggest that the low hardness of squid may indicate a poor network structure, which needs further investigation.

Scanning electron microscopy images provided structural insight into these textural differences (Figure 2i,j). The pork gel displayed a dense, continuous microstructure with minimal pores, indicative of a well-formed network. In contrast, the squid sample showed a loose, porous structure with noticeably larger cavities. In fermented silver carp mince, as fermentation progressed, microscopic results showed a higher density in samples due to increased interactions [25]. The loose structure in the squid samples is direct evidence of weak interactions. These findings collectively indicate that the sodium citrate-treated squid developed a weaker gel network than pork, consistent with its lower mechanical strength.

### 2.3. Comparison of Water State of Giant Squid and Pork

**Figure 3:**
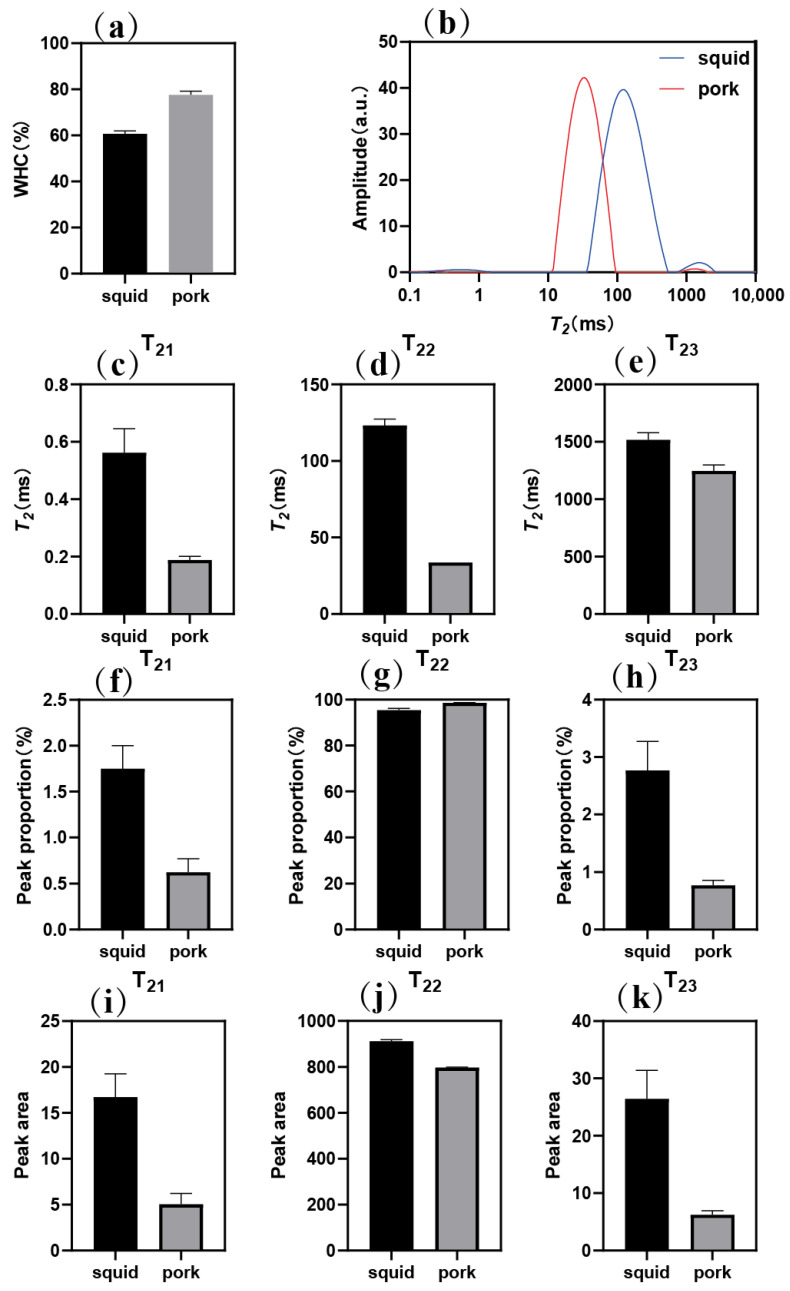
WHC (**a**), T_2_ relaxation time distribution (**b**), T_2_ relaxation time (**c**–**e**), peak proportion (**f**–**h**), and peak area (**i**–**k**) of giant squid and pork samples.

The water holding capacity (WHC) of the squid samples was about 60.7%, while that of the pork samples was about 77.6% (Figure 3a), significantly higher than the former (*p* < 0.05). WHC is an indicator of gel quality, describing the ability to hold water [26]. It is believed that a higher WHC indicates that a sample has formed a good network structure, which leads to more water retention [27]. LF-NMR was used to study the water distribution of the pork and squid samples. Figure 3b shows three peaks in the samples. Based on the literature, relaxation times of 0–10 ms (T_21_), 10–100 ms (T_22_), and 100–1000 ms (T_23_) can be assigned to bound water, immobilized water, and free water, respectively [28]. As illustrated in Figure 3c–e, compared with pork, the peaks at T_21_, T_22,_ and T_23_ of squid shifted to a higher relaxation time. Figure 3f–h shows the proportion of each peak in the samples. It can be seen that the proportion of T_21_ and T_23_ in squid was larger than that of pork. In terms of peak area, the T_21_, T_22_, and T_23_ areas of the squid were larger than those of the pork (Figure 3i–k). These results indicate that the squid gel not only contained more total water but also that this water was more mobile and less tightly bound, a characteristic often associated with a weak gel structure [29]. This aligns with the aforementioned results, confirming that the denser, more continuous network of the pork gel effectively immobilized water, whereas the coarse, porous structure of the squid gel led to poor water retention.

## 3. Conclusions

The present study confirmed that the gel quality of sodium citrate-treated giant squid was markedly inferior to that of pork. Specifically, the squid gel exhibited poorer rheological properties, a weaker microstructure, lower water-holding capacity, and inferior textural properties (e.g., a hardness of 259.78 g vs. 3305.92 g for pork), which collectively indicate an underdeveloped gel network. These results demonstrate that directly using soaked giant squid for surimi production is not advisable, and targeted interventions are necessary to improve the gel properties. The poor gel quality is likely attributable to the intrinsic properties of squid protein, potential quality deterioration during storage [6], and the impact of the soaking process itself [30]. To address this, future research should focus on methods to enhance gel formation. Promising strategies, as indicated by previous studies, include the addition of compounds such as organic acids [9] or laver powder [8], as well as the application of fermentation techniques [31,32]. Our prior work has laid a foundation by showing that co-inoculation with *Lacticaseibacillus casei* and *Staphylococcus carnosus* can significantly improve the gel quality of sodium citrate-treated giant squid samples supplemented with starch, soy protein isolate, and other ingredients [33,34]. Nevertheless, the fundamental reasons for the weak gelation of giant squid and the effect of different deodorization methods on its gel quality require further investigation.

## 4. Materials and Methods

### 4.1. Materials

The squid were obtained from Ningbo Feirun Co., Ltd. (Ningbo, China) and stored below −20 °C. The pork was purchased from a supermarket (Ningbo, China) and used on the same day.

### 4.2. Preparation of Squid and Pork Surimi

**Figure 4:**
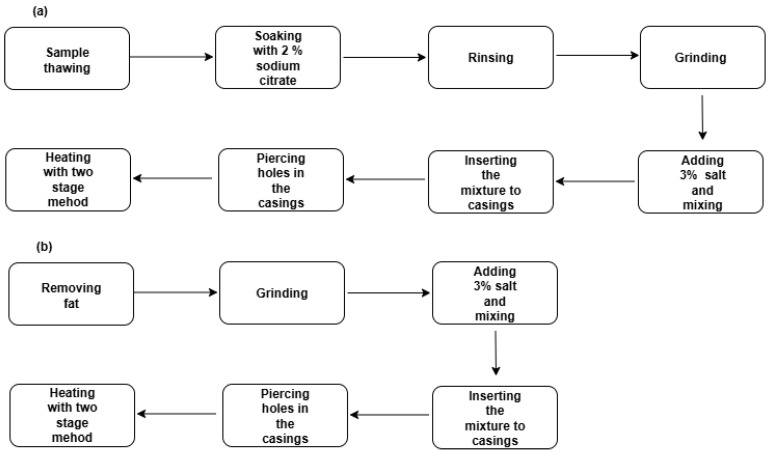
A flowchart of the steps for producing giant squid surimi (**a**) and pork surimi (**b**).

The steps for the preparation of squid and pork surimi are shown in Figure 4. The squid was thawed using running tap water and cut into small pieces. Then, the squid were soaked in 2% sodium citrate (dissolved in cool boiled water) (at a ratio of 1:3 (*w*:*v*)) for 15 h at 4 °C, as previously described [33]. After being washed with tap water, the squid were minced with a meat grinder and 3% salt was added. After mixing, the samples were stuffed into 30 mm collagen casings. After punching holes with toothpicks in the casings, the samples were cooked using a two-stage heating method as previously described (40 °C, 60 min; 90 °C, 30 min) [33]. Then, the samples were immersed in cool boiled water for 30 min at 4 °C and stored at 4 °C for further analysis [33]. The pork was minced after removing the fat. Subsequently, it was mixed with 3% salt and processed according to the method described above.

### 4.3. Rheology Measurements

The rheological characterization for each experimental group was conducted in triplicate measurements employing a rheometer (Discovery HR-2, TA Instruments, New Castle, DE, USA). The testing protocol was carried out at a constant temperature of 25 °C. The geometry was set with a fixed gap of 1000 μm. All measurements were performed under a constant strain of 0.1% while applying an oscillatory frequency sweep that ranged from 0.1 to 10 Hz.

### 4.4. Texture Profile Analysis (TPA)

TPA was performed using a texture analyzer (TA. XT Plus, Stable Micro System, Godalming, UK). The parameters of the TPA were identical to previous research [33]: the pre-test speed, test speed, and post-test speed were 1 mm/s, 1 mm/s, and 5 mm/s, respectively; target model was strain; strain was 30%; time was 5 s; and trigger force was 5 g. TPA tests were performed in quadruplicate.

### 4.5. Observation of Microstructure

The microstructure of the samples was examined according to a previously reported procedure with modifications [35]. Briefly, samples were first sectioned into small fragments and fixed in glutaraldehyde solution (2.5%) for 24 h (stored at 4 °C). After rinsing, we gradually removed water with increasing concentrations of ethanol, then rinsed with a mixture of ethanol and tert-butanol at different concentrations to remove the ethanol, and finally immersed the sample in a small amount of tert-butanol. After freeze drying, before imaging, the samples were sputter-coated with gold (E-1010, Hitachi Ltd., Tokyo, Japan) and observed under a scanning electron microscope (S–3400N, Hitachi Ltd., Tokyo, Japan).

### 4.6. Water-Holding Capacity (WHC) Analysis

WHC was measured after an overnight storage period, using a previously reported method with slight modifications [36]. Briefly, the samples were precisely weighed (recorded as W_1_), wrapped in filter paper, and centrifuged (10,000× *g*, 4 °C, 10 min) with a centrifuge (TGL-18M, Bioridge, Shanghai, China). After centrifugation, the samples were weighed again (W_2_). WHC was calculated as the ratio of W2 to W1 (W2/W1) and expressed as a percentage. WHC measurements were performed in triplicate for each group.

### 4.7. Low-Field Nuclear Magnetic Resonance (LF-NMR)

LF-NMR was measured on a nuclear magnetic resonance analyzer (NMI20-060H-1, Niumag, Suzhou, China). The parameters were as follows: number of echoes, 15,000; other parameters were identical to those in [34]. For each group, the T_2_ measurement was performed in quadruplicate, with each replicate scanned three times.

### 4.8. Statistical Analysis

Student’s *t*-test was performed using Statistical Package for the Social Sciences 19.0 (SPSS Inc., Chicago, IL, USA). *p* < 0.05 was regarded as statistically significant.

## Data Availability

The data may be obtained from the corresponding author upon reasonable request.

## References

[1] Ding H.C., Li X.P., Li R.Z., Yi S.M., Xu Y.X., Mi H.B., Li J.R. (2019). Changes of water state and gel characteristics of Hairtail (*Trichiurus lepturus*) surimi during thermal processing. J. Texture Stud..

[2] Jiang Q., Wu W., Han J., Chung H.Y., Gao P., Yu D., Yu P., Xu Y., Xia W. (2022). Characteristics of silver carp surimi gel under high temperature (≥100 °C): Quality changes, water distribution and protein pattern. Int. J. Food Sci. Technol..

[3] Lu W., Qin Y., Ruan Z. (2021). Effects of high hydrostatic pressure on color, texture, microstructure, and proteins of the tilapia (*Orechromis niloticus*) surimi gels. J. Texture Stud..

[4] Chen N., Gao P., Jiang Q., Yu X., Li P., Xu Y., Yu D., Yang F., Xia W. (2022). Effects of deheading and rinsing pretreatment on the quality of white leg shrimp (*Litopenaeus vannamei*) surimi based on endogenous proteases. Food Res. Int..

[5] Tolano-Villaverde I.J., Santos-Sauceda I., Santacruz-Ortega H., Ramírez-Wong B., Brown-Bojórquez F., Suárez-Jiménez G.M., Márquez-Ríos E. (2020). Evaluation of the gelling ability of actomyosin-paramyosin from giant squid mantle (*Dosidicus gigas*). Int. Food Res. J..

[6] Gómez-Guillén M.C., Montero P., Solas M.T., Borderías A.J. (1998). Thermally induced aggregation of giant squid (*Dosidicus gigas*) mantle proteins. physicochemical contribution of added ingredients. J. Agric. Food Chem..

[7] Sánchez-Brambila G.Y., Alvarez-Manilla G., Soto-Cordova F., Lyon B.G., Pacheco-Aguilar R. (2004). Identification and characterization of the off-flavor in mantle muscle of jumbo squid (*Dosidicus gigas*) from the Gulf of California. J. Aquat. Food Prod. Technol..

[8] Yang R., Xu A., Chen Y., Sun N., Zhang J., Jia R., Huang T., Yang W. (2020). Effect of laver powder on textual, rheological properties and water distribution of squid (*Dosidicus gigas*) surimi gel. J. Texture Stud..

[9] Chu Y., Deng S., Lv G., Li M., Bao H., Gao Y., Jia R. (2022). Improvement of gel quality of squid (*Dosidicus gigas*) meat by using sodium gluconate, sodium citrate, and sodium tartrate. Foods.

[10] Niu F., Li X., Lin C., Hu X., Zhang B., Pan W. (2025). The mechanism of egg white protein to enhance the thermal gel properties of giant squid (*Dosidicus gigas*) surimi. Food Chem..

[11] Moreno H.M., Cardoso C., Solas M.T., Borderías A.J. (2009). Improvement of cold and thermally induced gelation of giant squid (*Dosidicus gigas*) surimi. J. Aquat. Food Prod. Technol..

[12] Campo-Deaño L., Tovar C.A., Jesús Pombo M., Teresa Solas M., Javier Borderías A. (2009). Rheological study of giant squid surimi (*Dosidicus gigas*) made by two methods with different cryoprotectants added. J. Food Eng..

[13] Yang W., Chen S., Yu S., Zhang Y. (2019). Effects of different soaking processes and additives on the acid removal efficiency of squid. Jiangxi Fish. Sci. Technol..

[14] Zhou X., Kang M. (2015). Deacidification of Peru squid. Food Sci. Technol..

[15] Zhao H., Xu Y., Li X., Zhu W., Yi S., Li J., Li Y. (2019). Effect of ultrasonic-assisted deacidification on quality of *Dosidicus gigas*. Food Res. Dev..

[16] Yu Z., Gao Y., Wu M., Zhao C., Liu X., Zhang X., Zhang L., Chen Y. (2024). Effects of phosphorylated ovalbumin on the quality of pork myofibrillar protein gel: An insight into gelling and physicochemical properties. J. Futur Foods.

[17] Ji L., Zhou Y., Nie Q., Luo Y., Yang R., Kang J., Zhao Y., Zeng M., Jia Y., Dong S. (2024). The potential correlation between bacterial diversity and the characteristic volatile flavor compounds of Sichuan sauce-flavored sausage. Foods.

[18] Chen X., Yan F., Qu D., Wan T., Xi L., Hu C.Y. (2024). Aroma characterization of Sichuan and Cantonese sausages using electronic nose, gas chromatography–mass spectrometry, gas chromatography-olfactometry, odor activity values and metagenomic. Food Chem. X.

[19] Shi L., Wang Q., Xie Z., Wu C., Peng T., Nian X., Li L., Li H., Chen T. (2024). The changes of fungal community and flavor substances in Yunnan-style sausages: A comparative analysis of different drying methods. Food Chem..

[20] Ye Y., Chen F., Shi M., Wang Y., Xiao X., Wu C. (2024). Gel properties and protein structures of minced pork prepared with κ-carrageenan and non-meat proteins. Gels.

[21] Li H., Sheng W., Nunekpeku X., Li C., Zhang W., Wang Y. (2025). Mechanistic insights into gel quality changes in pork batters induced by ultrasound: From myofibrillar protein aggregation to conformation. Int. J. Biol. Macromol..

[22] Zhang Y., Guo X., Xiong H., Zhu T. (2022). Effect of modified soy protein isolate on dough rheological properties and noodle qualities. J. Food Process. Preserv..

[23] Xu X., Cui H., Yuan Z., Xu J., Li J., Liu J., Liu H., Zhu D. (2022). Effects of different combinations of probiotics on rheology, microstructure, and moisture distribution of soy materials-based yogurt. J. Food Sci..

[24] Khatkar A.B., Kaur A. (2018). Effect of protein incorporation on functional, thermal, textural and overall quality characteristics of instant noodles. J. Food Meas. Charact..

[25] Xu Y., Xia W., Yang F., Nie X. (2010). Protein molecular interactions involved in the gel network formation of fermented silver carp mince inoculated with *Pediococcus pentosaceus*. Food Chem..

[26] Liao J., Shi H., Wang J., Xia G., Zhao Y., Yu G., Shen X. (2025). Investigation of the gel properties and gelation mechanism of a surimi blend composed of skipjack tuna (*Katsuwonus pelamis*) and purpleback flying squid (*Symplectoteuthis oualaniensis*). Foods.

[27] Huang X., Liu Q., Wang P., Song C., Ma H., Hong P., Zhou C. (2024). Tapioca starch improves the quality of *Virgatus nemipterus* surimi gel by enhancing molecular interaction in the gel system. Foods.

[28] Liu Y., Sun Q., Pan Y., Wei S., Xia Q., Liu S., Ji H., Deng C., Hao J. (2021). Investigation on the correlation between changes in water and texture properties during the processing of surimi from golden pompano (*Trachinotus ovatus*). J. Food Sci..

[29] Pan J., Jia H., Shang M., Xu C., Lian H., Li H., Dong X. (2018). Physiochemical properties and tastes of gels from Japanese Spanish mackerel (*Scomberomorus niphonius*) surimi by different washing processes. J. Texture Stud..

[30] Jin M., Zhou Y., Xu Y., Tang J., Yang W., Xu P. (2012). Changes of biochemical properties of muscle protein during the process of *Dosidicus gigas* frozen goods. Sci. Technol. Food Ind..

[31] Xu Y., Xia W., Yang F., Kim J.M., Nie X. (2010). Effect of fermentation temperature on the microbial and physicochemical properties of silver carp sausages inoculated with *Pediococcus pentosaceus*. Food Chem..

[32] Hu Y., Xia W., Ge C. (2007). Effect of mixed starter cultures fermentation on the characteristics of silver carp sausages. World J. Microbiol. Biotechnol..

[33] Mu H., Weng P., Wu Z. (2023). Effect of inoculation with *Lacticaseibacillus casei* and *Staphylococcus carnosus* on the quality of squid (*Dosidicus gigas*) surimi sausage. Fermentation.

[34] Mu H., Weng P., Wu Z. (2025). Mixed inoculation with *Lacticaseibacillus casei* and *Staphylococcus carnosus* improves safety, gel properties and flavor of giant squid surimi without added seasonings. Fermentation.

[35] Mi J., Ni W., Huang P., Hong J., Jia R., Deng S., Yu X., Wei H., Yang W. (2022). Effect of acetylated distarch adipate on the physicochemical characteristics and structure of shrimp (*Penaeus vannamei*) myofibrillar protein. Food Chem..

[36] Yu X., Wang Y., Xie Y., Wei S., Ding H., Yu C., Dong X. (2022). Gelation properties and protein conformation of grass carp fish ball as influenced by egg white protein. J. Texture Stud..

